# Preliminary Results of Cryoablation for Surgical Treatment of Arrhythmias in Adults With Congenital Heart Disease

**DOI:** 10.3389/fcvm.2021.770221

**Published:** 2021-12-03

**Authors:** Giulia Poretti, Stiljan Hoxha, Antonio Segreto, Gardellini Jacopo, Camilla Sandrini, Giuseppe Faggian, Alessandro Varrica, Massimo Chessa, Alessandro Giamberti, Giovanni Battista Luciani

**Affiliations:** ^1^Division of Cardiac Surgery, Department of Surgery, Dentistry, Paediatrics and Gynecology, University of Verona School of Medicine, Verona, Italy; ^2^Division of Cardiology, Department of Medicine, University of Verona School of Medicine, Verona, Italy; ^3^Department of Congenital Cardiac Surgery, IRCCS Policlinico San Donato, San Donato Milanese, Italy; ^4^Department of Congenital Cardiology, IRCCS Policlinico San Donato, San Donato Milanese, Italy

**Keywords:** congenital heart surgery, arrhythmias, cryoablation, adult congenital heart disease, complex congenital heart disease

## Abstract

**Background:** Arrhythmias in adult congenital heart disease (ACHD) are responsible for the majority of hospital admissions and 20–25% of late deaths. Since need for further cardiac operations is frequent in ACHD, concomitant arrhythmia surgery represents a strategic treatment modality.

**Material and Methods:** A two-center retrospective study was undertaken on cryoablation of supraventricular arrhythmias in 25 conescutive ACHD patients (16/9, M/F, median age 38.5 years, IQR 38–60) operated between 01/2017 and 12/2020. Nineteen (76%) had undergone one or more previous cardiac operations and 8 (32%) one or more trans-catheter ablations. Indications included Fontan conversion in seven patients, septal defect repair in 6, pulmonary valve replacement in 10 and tricuspid surgery in 2. Open-heart cryoablation included: 4 cavotricuspid isthmus ablations, 19 right-sided Maze for atrial tachycardia/flutter, and 2 Cox-Maze III for atrial fibrillation.

**Results:** There were 2 (8%) hospital deaths, unrelated to cryoablation, due to low cardiac output syndrome. There were no intraoperative complications related to cryoablation. Seven (28%) patients required pace-maker implantation due to post-operative atrioventricular block. All patients were discharged on oral antiarrhythmic and anticoagulantion for 6 months. After a median follow-up of 14 months (IQR 7–27) no late mortality was observed. At follow-up, 16/23 (69%) patients are in stable sinus rhythm, 12 without anti-arrhythmic therapy. Two (8.6%) patients had relapse of arrhythmia. Freedom from arrhythmia was 90.9% and cumulative risk of recurrence was 9.6%.

**Conclusions:** Intraoperative cryoablation is safe and effective procedure. Surgical treatment of arrhythmias should always be considered in ACHD, whenever further open-heart repair is needed.

## Introduction

Adults with congenital heart disease (ACHD) are a growing population. Due to medical, surgical, and technological evolutions over the past decades, more than 90% of patients with congenital heart disease (CHD) are currently surviving to adulthood and the number of ACHD (2, 3 million in Europe) now far exceeds the number of children (1, 2). Arrhythmias are the most common cardiologic reason for admission to the hospital and a significant source of morbidity and mortality in this population (3). Arrhythmias have a multifactorial etiology: part of the congenital heart defect, consequence of surgery or result of hemodynamic abnormalities (4). In the past years different techniques have been tested for surgical treatment of arrhythmias. Starting from the original Cox Maze procedure, several energy sources have been used to replace the traditional “cut-and-sew.” In this study we report our prelimary experience of intraoperative cryoablation of supraventricular arrhythmias in ACHD undergoing cardiac surgery.

## Materials and Methods

This is a two-Center retrospective study. Between January 2017 and December 2020, 25 consecutive ACHD patients with supraventricular arrhythmias underwent open heart surgery with concomitant cryoablation. All patients signed informed consent for both structural heart surgery and cryoablation.

### Surgical Methods

All operations were done through a median sternotomy using total cardiopulmonary bypass and cardioplegic arrest. A cryosurgical probe *(cryoFORM*^®^ *probe, AtriCure Inc., Mason, OH, USA)* was used in all patients for linear lesion and ablation lines. Multiple cryoablation lesions were placed at a temperature of −60°C for 90 s. Lesion and ablation lines have been drawn according to previously described techniques (5). Right atrial appendage was routinely excised. Right atrial incisions were from the amputated right appendage toward the crista terminalis, and from the middle of the right atriotomy toward the inferior part of the atrium. Right atrial ablation lines were as follows: from the superior vena cava (SVC) toward inferior vena cava (IVC), from the excised right atrial appendage to the tricuspid valve annulus, from the crista terminalis to the ASD or fossa ovalis, between the IVC to the coronary sinus and between the IVC and tricuspid valve annulus. In left atrium the ablation lines were: around the four pulmonary veins, between the left and right pulmonary veins, between the base of the left appendage and the pulmonary veins, between the pulmonary veins and the mitral valve annulus, connecting the middle of the line toward the mitral valve annulus and the left atrial surface of the coronary sinus roof. There was no intraoperative electrophysiological evaluation of the ablation lines.

### Statistical Analysis

Categorical variables were reported as percentage; continuous as median and interquartile range (IQR). Endpoints of the study were to verification of the efficacy, in terms of freedom from recurrence of an arrhythmic event, and safety, in terms of mortality and complications, of surgical cryoablation.

Freedom from arrhythmia recurrence and cumulative risk of recurrence were investigated using Kaplan–Meier actuarial survival curves.

## Results

### Patient Population

There were 9 females and 16 males with a median age of 38.5 years (IQR 38–60). Seven patients had univentricular heart (UVH) (tricuspid atresia in 3 patients, double inlet left ventricle with pulmonary stenosis in 3 and congenitally corrected transposition of great arteries with mitral atresia in 1), 8 patients had severe pulmonary valve regurgitation (6 after Tetralogy of Fallot repair and 2 after repair of right ventricular outflow tract obstruction), 1 right ventricular outflow tract obstruction (after Tetralogy of Fallot repair), 2 atrio-ventricular septal defects (1 complete and 1 intermediate), 4 had an atrial septal defect (ASD) (1 ostium secundum type ASD, 2 sinus venosus ASD, and 1 associated with partial anomalous pulmonary venous return), 1 had ventricular septal defect with tricuspid regurgitation and ASD and 2 had tricuspid regurgitation (1 Ebstein anomaly and 1 after Tetralogy of Fallot repair).

Nineteen patients had history of previous open heart surgery: 7 had undergone an atrio-pulmonary Fontan operation, 8 Tetralogy of Fallot repair, 1 infundibular patch for right ventricular outflow tract obstruction, 2 repair of atrio-ventricular septal defect, and 1 VSD and ASD closure associated with tricuspid valve repair.

All patient had supraventricular arrhythmias: 9 patients had atrial tachycardia, 5 atrial fibrillation (2 permanent), 7 atrial flutter (5 permanent), and 4 alternating atrial fibrillation and atrial flutter.

Pre-operative ejection fraction (EF) was normal in 13 patients, mildly reduced in 8 (50 > EF > 50%) and moderately reduced (45 > EF < 50%) in 4 patients.

Thirteen patients were in New York Heart Association (NYHA) functional class III (5 patients with UVH, 6 with severe pulmonary insufficiency, 1 with Ebstein anomaly, and 1 with atrio-ventricular canal), 1 patient was in NYHA class I and the remaining 11 patients were in NYHA class II.

Thirteen patients had a pre-operative antiarrhythmic therapy and 8 had history of previous unsuccessful catheter ablation. Arrhythmias had a median duration of 5 years (IQR 1–7) before surgical ablation. More than half of the patients (6) had arrhythmia for more than 3 years, which is a known risk factor for surgical ablation failure (5). Pre-operative data are summarized in Table 1.

**Table 1:** Demographics and baseline characteristics of patients.

Male gender	16
Age (years), median (range)	38.5 (38–60)
Palliative surgery	7
Reintervention	19
Number of previous operation	1 (1,2)
Height (cm), median (range)	170 (166–175)
Weight (kg), median (range)	73 (62–80)
Previous transcatdeter ablation	8
Previous anti-arrhytdmic tderapy	13
Pre-operative NYHA class	
I	1
II	11
III	13
IV	0
LVEF or UVEF (%), median (range)	54 (50–58)
Pre-operative arrhytdmia	
Atrial tachycardia	9
Atrial flutter	7
Atrial fibrillation	5
Atrial fibrillation/atrial flutter	4
Years of arrhytdmia, median (range)	5 (1–7)

*NYHA, New York Heart Association; LVEF, left ventricular ejection fraction; UVEF, univentricular ejection fraction*.

### Operative Data

Median time of aortic cross clamp was 61.5 min (IQR: 46–83). Primary surgical repair was performed on 6 patients: 1 tricuspid valve replacement, 1 pulmonary valve replacement, and 4 ASD repair (1 with partial anomalous pulmonary venous return). Associated surgical procedures in this group of patients were: right atrial free wall reduction in 1 patients, ASD/PFO closure in 2, and tricuspid valve repair in 2. Nineteen patients had a redo operation: Fontan conversion to total extracardiac cavo-pulmonary connection in 7 cases, pulmonary valve implantation in 8, tricuspid valve replacement in 2, mitral valve replacement in 1, and mitral valve repair in 1. Associated cardiac surgical procedures were: tricuspid valve repair in 6 patients, atrial-septectomy in 5, left atrial appendage closure in 2, right ventricular outflow tract remodeling in 2, mitral valve repair in 1, ASD repair in 1, aortic valve replacement in 1, and right atrial free wall reduction in 1. In 9 patients (7 Fontan conversion), epicardial leads for permanent pacing were placed at the end of operation.

All patients underwent surgical ablation. Pattern of ablation was chosen in accordance with the type of arrhythmia. In common atrial flutter, a linear lesion connecting the inferior vena cava and coronary sinus to the tricuspid valve was performed (*n* = 4) (cavo-tricuspid isthmus ablation). We performed right-sided Maze procedure (*n* = 19) for atrial tachycardia and Cox Maze III procedure (*n* = 2) (right and left atrium) for atrial fibrillation. However, only two Cox Maze III procedures were performed because in the remaining cases of atrial fibrillation (3 patients) there were technical issues with ablation of the left atrium.

Patients diagnosis, type of arrhythmia, type of ablation and cardiac intervention are summarized in Table 2.

**Table 2:** Type of arrhythmia and surgical procedure.

**CHD**	**Type of arrhythmia**	**Ablation site**	**Type of operation**	**Associated procedures**
UVH *n* = (7)	AT 5	RA	Conversion Fontan 7	PMK 5 MVR 1 AS 3
	AF 1	RA		PMK 1 AS 1
	AFl 1	RA		PMK 1 AS 1
PVR (8)	AFl 4	RA	Pulmonary valve implantation 8	TVR 3 Left appendage closure 1 ASD closure 1 Right ventriculoplasty 2
	AT 1	RA		AVR 1 TVR 1
	AF+AFl 2	CTI-RA		TVR 1
	AF 1	RA		TVR 1 Left appendage closure 1 ASD closure 1
PVS (1)	AFl	RA	Pulmonary valve implantation 1	
ASD (4)	AF2	Cox-Maze III-RA	ASD closure 2	Left appendage closure 1
	AFl 1	CTI	PAPVC correction 1	PMK 1
	AF + AFl 1	RA	ASD colosure 1	TVR 1
AVC (2)	AF 1	Cox-Maze III	MVR	Left appendage closure 1
	AF + AFl 1	CTI	MVI	TVR 1
VSD + ASD (1)	AT	RA	TVI	PMK 1
				RA free wall reduction
TVR (Ebstein anomaly and ToF) (2)	AT 2	CTI	TVI 2	PMK 1
		RA		RA free wall reduction 1

*UVH, univentricualr heart; PVR, pulmonary valve regurgitation; PVS, pulmonary valve stenosis; ASD, atrial septal defect; AVC, atrio-ventricular canal; VSD, ventricular septal defect; TVR, tricuspid valve regurgitation; ToF, Tetralogy of Fallot; AT, atrial tachycardia; AF, atrial fibrillation; AFl, atrial flutter; RA, right atrium; CTI, cavo-tricuspid isthmus; PAPVC, partial anomalous pulmonary venous connection; MVR, mitral valve repair; MVI, mitral valve implantation; TVI, tricuspid valve implantation; TVR, tricuspid valve repair; PMK, pacemaker epicardial wires; AS, atrioseptostomy; AVR, aortic valve replacement*.

### Clinical Outcome

There were two hospital deaths, unrelated to cryoablation, due to worsening low cardiac output syndrome, after tricuspid replacement for Ebstein anomaly and after Fontan conversion, each in 1 patient.

The first patient was a 44-year-old man with Ebstein's anomaly resulting in severe right atrial and right ventricular dilatation with right moderate ventricular dysfunction and severe tricuspid regurgitation. The patient also suffered from HIV immunodeficiency with previous cerebral infection and Kaposi's Sarcoma. In the post-operative period he suffered from severe right ventricular dysfunction with low cardiac output syndrome (LCOS), which required ECMO support.

The second patient died was a 39-year-old female with univentricular heart (tricuspid atresia), who had had previously two cardiac surgeries (the last was atrio-pulmonary Fontan, 33 years earlier). The patient suffered from a serious right atriomegaly with frequent episodes of atrial tachycardia, so she was a candidate for Fontan conversion surgery. Pre-operative ventricular function was mildly reduced (50%). In the post-operative, the patient suffered from severe single ventricle dysfunction resulting in LCOS and the need for ECMO implantation. Both patients could not be weaned from ECMO due to multi-organ failure.

Seven patients required pace-maker implantation due to post-operative sinus node dysfunction or atrioventricular conduction abnormalities. All patients were discharged on oral antiarrhythmic for 3–6 months and anticoagulants for 6 months. At discharge, 15 patients were in sinus rhythm, 5 had a stable pacemaker rhythm, 2 had atrial fibrillation, and 1 atrial flutter. One patient discharged in sinus rhythm had a pacemaker implant 5 months after the operation due to the presence of sinus node dysfunction.

During a median follow-up of 14 months (IQR 7–27), there was no late mortality and 17/23 patients had an improvement of NYHA functional class. Five patients in NYHA III progressed to class II and 4 to class I; eight patients progressed from class II to class I.

At follow up electrocardiogram, 16 patients were in sinus rhythm, 6 with stable pacemaker rhythm, and 1 with permanent atrial fibrillation.

**Figure 1:**
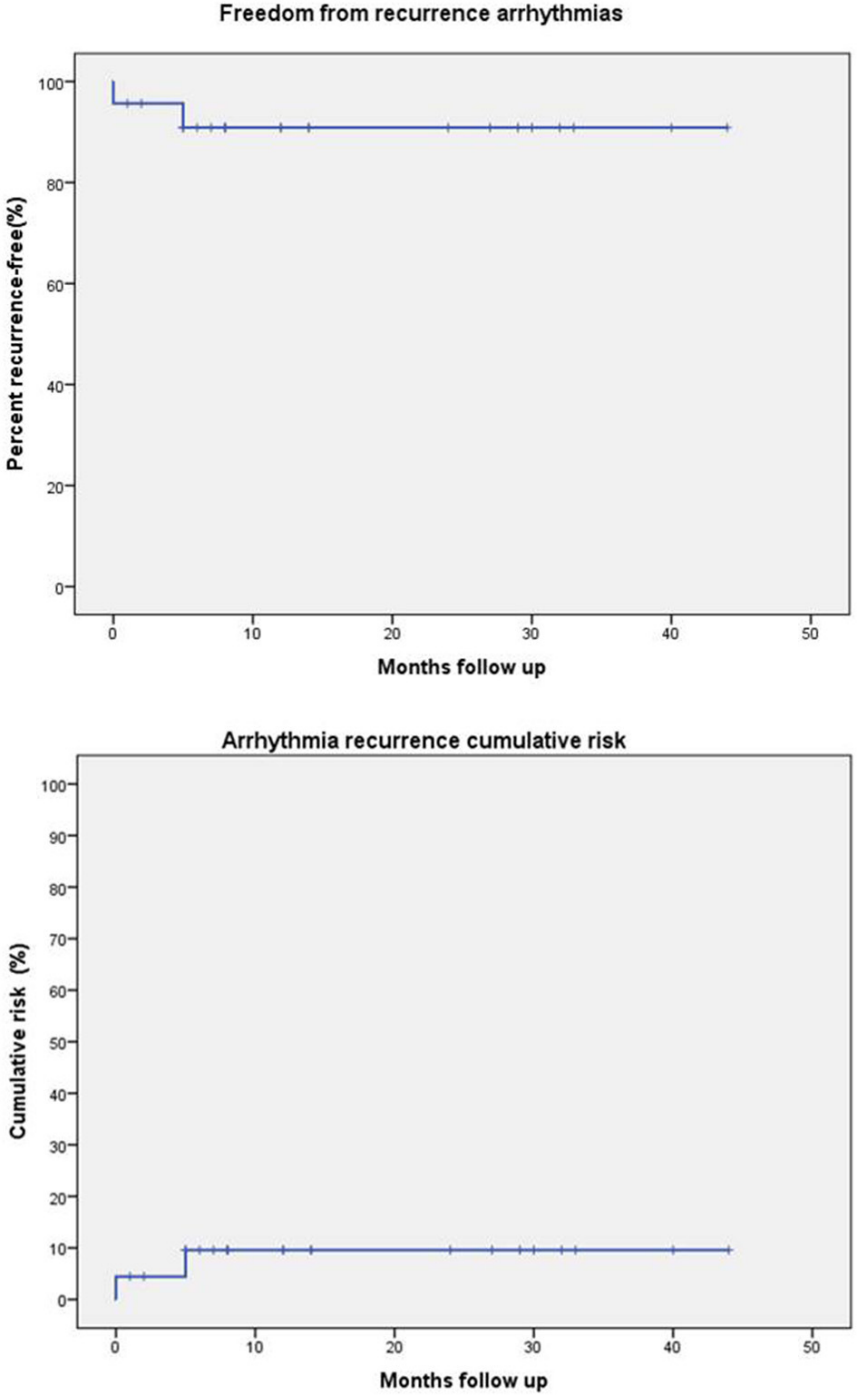
Freedom from recurrence of arrhythmia and cumulative risk of recurrence.

Recurrence of arrhythmia occurred in 2/23 (8.6%) patients, more than 3 months after surgery. These patients presented at surgical ablation with history of atrial fibrillation lasting 4 and 19 years, respectively, and both had atrial fibrillation, which was treated with right-sided Maze rather than Cox maze III due to technical issues. Sixteen (69%) patients are in stable sinus rhythm, 12 without any anti-arrhythmic therapy. At median follow up of 14 months (IQR 7–27), freedom from recurrence of arrhythmia was 90.9% and cumulative risk of recurrence was 9.6% (Figure 1).

## Discussion

Arrhythmias are an emerging and challenging problem in ACHD, since they represent the primary cause for hospital admission and a leading cause of morbidity and mortality. Arrhythmias not only cause unsettling symptoms, thereby compromising the patient's quality of life, but can also lead to or worsen heart failure. Furthermore, both supraventricular and ventricular arrhythmias may be associated with the risk of sudden cardiac death (7). The substrate of the arrhythmia correlates with structural abnormalities due to congenital defects (i.e., Ebstein anomaly and congenitally corrected transposition of great arteries) and with the surgical scars, as well as materials used for the repair, which can create pro-arrhythmic foci. In addition, pathological hemodynamic conditions can lead to arrhythmia through dilation, hypertrophy, and fibrosis of cardiac chambers. The incidence and substrate of arrhythmias are directly related: the more complex the substrate, the earlier and more often arrhythmias occur.

In patients with atrial septal defect (ASD), atrial arrhythmias occur more frequently due to the volume load of the right atrium alone. Without surgical repair, 10–15% of patients develop atrial flutter and/or atrial fibrillation by the age of 40 and 20–40% by the age of 60 (8, 9). The incidence of arrhythmias increases with age also in patients with repaired tetralogy of Fallot, from 3.3% in those aged 20–30 to 17% in those over 50 (10).

The incidence of atrial tachycardia after the Fontan operation approaches 30–50% after 5 years follow-up. In single ventricle patients, atrial arrhythmias are not only due to previous surgical manipulation of atrial cavities, but more importantly, these are linked to long standing after-load mismatch inherent with single ventricle physiology and the attendant chronic ventricular hypertrophy. This pathogenesis is suggested by the observation that extracardiac conduit total cavo-pulmonary connection, a very widely adopted modification of the lateral tunnel Fontan, has not been able to free single ventricle patients from the burden of arrhythmias. Atrial arrhythmias in Fontan patients are very poorly tolerated as they can lead to severe heart failure with low cardiac output and to a significant reduction in exercise tolerance (11).

Longer life expectancy and exposure to conventional risk factors further increased the prevalence of arrhythmias in ACHD, that has reached 50% (4, 7).

In consideration of the increasing incidence of arrhythmias and the severe adverse effects in this patient population, treatment of arrhythmias is one of the key challenges in the treatment of ACHD. Anti-arrhythmic strategies include conventional medical therapy, catheter ablation, and surgical therapy. Conventional medical treatment is not always effective in controlling arthymias in ACHD (12). This observation is confirmed by the present experience, as 52% of our patients had pre-operative antiarrhythmic drugs which were not effective. Moreover, 8 of our patients (32%) presented history of catheter ablations, which also were ineffective. Surgical ablation should always be considered in patients with associated structural cardiac defects requiring surgery.

In the past, surgical correction or palliation of the congenital heart defect *per se* was considered curative for the treatment of the arrhythmia. Nowadays, it has been suggested by several studies that arrhythmia surgery in addition to conventional surgery is instrumental in achieving more satisfactory results (8, 13, 14).

Historically, several techniques have been tested for the surgical treatment of arrhythmias. Starting from the original Cox Maze procedure, several energy sources have been used to replace the traditional “cut-and-sew.” Among these, cryoablation aroused particular interest for its many advantages: the most time-tested safety record, low risk of bleeding, perforation, or collateral damage (collagen tissue and vasculature are unaffected), reduced endocardial thrombus formation and ease of use with extra 10–20 min operating room time added per atrium (6). The present is the largest experience to date with a novel device *cryoFORM® (CF) probe* (AtriCure Inc., Mason, OH, USA) for intra-operative, open atrial cryoablation in ACHD. We adopted three different types of ablation according to the type of arrhythmia. For common atrial flutter we use the ablation of the cavotricuspid isthmus because is well-known that this arrhythmia is related to a macroreentry circuit between the annulus of the tricuspid valve and the orifice of the inferior vena cava (15). In presence of atrial tachycardia, we performed a right-sided maze procedure. According to recent recommendations for management of arrhythmias in ACHD, an extended right maze has greater efficacy than ablation of the isthmus alone, especially in ACHD in which supraventricular arrhythmias are often due to complex reentry circuits (16).

Finally, for atrial fibrillation most recent studies recommend performing a Cox Maze III procedure, with ablation of both the left atrium and the right atrium. Ablation of the right atrium alone leads to a much higher incidence of recurrence (16). Therefore, whenever technically possible, we have used this approach in patients with pre-operative atiral fibrillation. The major complication observed herein was need for permanent pacing due to sinus node or atrioventricular node dysfunction, occurring days or months after the operation, despite the patient left the operating room with sinus rhythm. For this reason, all patients with Fontan conversion underwent an implantation of definitive epicardial wires, as the heart chambers were no longer reachable by the conventional transvenous access. Whereas, prevalence of permanent pacing reaching 20% of patients in this series represents a concerning finding, physiological properties associated with recovery of atrioventricular electro-mechanical coupling far outweigh disadvantages associated with permanent pacing. Most of the patients had recurrence of supraventricular arrhythmia in the peri-operative period, a frequent complication already highlighted in other studies and which does not compromise the medium and long-term outcome (17).

Our mid-term results are encouraging, showing a freedom from recurrence of arrhythmia >90%. Due to the limited patient population, it is difficult to isolate factors associated with recurrence of supraventricular arrhythmias. However, the cases of relapse observed occurred in patients suffering from atrial fibrillation treated with right-sided Maze (not with Cox-Maze III due to technical issues related to prior adhesions) and who had arrhythmias for longer than 3 years prior to surgery. Both variables were previously recognized as risk factors for arrhythmia recurrence after surgical ablation (5).

## Limitations of the Study

The present study suffers from some evident limitations, including the retrospective nature of the design, the small, and heterogeneous patient population and the short-term follow-up. Nonetheless, it is to date the largest report of its kind on intra-operative cryoablation during surgery for CHD in adult patients.

## Conclusions

The present experience suggests that intraoperative treatment of supraventricular arrhythmias by cryoablation associated with primary or reoperative repair of congenital heart disease is feasible, safe, and the midterm results are encouraging.

## Data Availability Statement

The original contributions presented in the study are included in the article/supplementary materials, further inquiries can be directed to the corresponding author.

## Ethics Statement

The studies involving human participants were reviewed and approved by Comitato Etico Azienda Ospedaliera Universitaria Integrata di Verona. Written informed consent for participation was not required for this study in accordance with the national legislation and the institutional requirements.

## Author Contributions

GP and SH drafted the manuscript. GL and AG designed the study. GP, SH, AS, GJ, CS, AV, and MC were responsible for the collection of data or analysis. GL, AG, and GF revised the manuscript. All authors read and approved the final manuscript.

## Conflict of Interest

The authors declare that the research was conducted in the absence of any commercial or financial relationships that could be construed as a potential conflict of interest.

## Publisher's Note

All claims expressed in this article are solely those of the authors and do not necessarily represent those of their affiliated organizations, or those of the publisher, the editors and the reviewers. Any product that may be evaluated in this article, or claim that may be made by its manufacturer, is not guaranteed or endorsed by the publisher.
